# Predicting Early Bulbar Decline in Amyotrophic Lateral Sclerosis: A Speech Subsystem Approach

**DOI:** 10.1155/2015/183027

**Published:** 2015-06-02

**Authors:** Panying Rong, Yana Yunusova, Jun Wang, Jordan R. Green

**Affiliations:** ^1^Department of Communication Sciences and Disorders, MGH Institute of Health Professions, 36 First Avenue, Boston, MA 02129, USA; ^2^Department of Speech-Language Pathology, University of Toronto, 160-500 University Avenue, Toronto, ON, Canada M5G 1V7; ^3^Department of Bioengineering, University of Texas at Dallas, 800 W. Campbell Road, Richardson, TX 75080, USA; ^4^Callier Center for Communication Disorders, University of Texas at Dallas, 1966 Inwood Road, Dallas, TX 75235, USA

## Abstract

*Purpose*. To develop a predictive model of speech loss in persons with amyotrophic lateral sclerosis (ALS) based on measures of respiratory, phonatory, articulatory, and resonatory functions that were selected using a data-mining approach. *Method*. Physiologic speech subsystem (respiratory, phonatory, articulatory, and resonatory) functions were evaluated longitudinally in 66 individuals with ALS using multiple instrumentation approaches including acoustic, aerodynamic, nasometeric, and kinematic. The instrumental measures of the subsystem functions were subjected to a principal component analysis and linear mixed effects models to derive a set of comprehensive predictors of bulbar dysfunction. These subsystem predictors were subjected to a Kaplan-Meier analysis to estimate the time until speech loss. *Results*. For a majority of participants, speech subsystem decline was detectible prior to declines in speech intelligibility and speaking rate. Among all subsystems, the articulatory and phonatory predictors were most responsive to early bulbar deterioration; and the resonatory and respiratory predictors were as responsive to bulbar decline as was speaking rate. *Conclusions*. The articulatory and phonatory predictors are sensitive indicators of early bulbar decline due to ALS, which has implications for predicting disease onset and progression and clinical management of ALS.

## 1. Introduction

Amyotrophic lateral sclerosis (ALS) is a progressive neurological disease defined by the degeneration of both upper and lower motor neurons in the brain and spinal cord. ALS is often subtyped into several variants based on the site of onset (e.g., bulbar, spinal, and respiratory [1]). Bulbar ALS, which affects speech and swallowing, is characterized by the eventual loss of speech intelligibility and ability to swallow [2–4]. The critical role of bulbar motor function on the quality of life and survival [5, 6] motivates the search for sensitive and specific markers of bulbar disease onset and progression.

The current standard assessment of bulbar function includes clinician-based estimates of speech intelligibility and speaking rate. Despite its widespread clinical use, speech intelligibility is not responsive to early phases of the disease; changes in speech intelligibility occur late in the disease course and long after the onset of bulbar motor symptoms [4, 7–12]. The slowing of speech, however, appears to precede declines in speech intelligibility, which tends to decline rapidly once speaking rate slows to approximately 120 words per minute (WPM) [4, 11, 13]. Therefore, the slowing of speaking rate to 120 WPM marks the onset of the rapid decline phase of speech intelligibility (i.e., intelligibility < 85%). In contrast to the normal speech phase (i.e., intelligibility ranged within 100~97%) and the slow decline phase (i.e., intelligibility ranged within 96%~86%), which correspond to minimal or slow declines in intelligibility, the rapid decline phase is characterized by precipitous declines in intelligibility and the eventual loss of speech communication within a short time span [3, 4].

Speaking rate as an early clinical marker of bulbar involvement, however, may be limited because talkers with mild oromotor weakness have a variety of ways (e.g., by reducing the magnitude of speech movement) to maintain a normal speaking rate [10]. In addition, speaking rate may be significantly impacted by losses in articulatory function while only minimally affected by losses in the resonatory and phonatory functions [14, 15]. Therefore, measures of bulbar impairment that rely on speaking rate may be insensitive to cases of ALS that are characterized by resonatory or phonatory impairment onset. In this study, we take a “subsystem” approach and explore the possibility that measures from the four major speech subsystems (i.e., respiratory, phonatory, articulatory, and resonatory) are more responsive to the early stages of bulbar deterioration than are current standard assessments, the system-level measures of speech intelligibility and speaking rate.

Previous studies have identified several promising candidate markers of bulbar motor decline using instrument-based measures of speech subsystem performance. Kent et al. [16] measured the physiologic function of the respiratory subsystem and identified declines in maximum ventilatory volume and vital capacity as primary indicators of respiratory decline related to bulbar ALS (see also [17–19]). Ramig et al. [20] studied the acoustic features related to the phonatory subsystem function and identified increased phonatory instability (e.g., increased variability in the amplitude and fundamental frequency [F0] of voice, increased jitter and shimmer, etc.) and reduced phonatory limits (e.g., F0 range) as acoustic indicators of vocal involvement (see also [16, 21–26]). Kelhetter [27] examined the resonatory subsystem function using aerodynamic measures and found that increased nasal airflow leakage during oral consonants was characteristic of the speech produced by patients with ALS (see also [21, 28]). Weismer et al. [29, 30] used acoustic analysis to infer about the articulatory differences between healthy controls and persons with ALS, which were observed in the temporal features of speech (e.g., total utterance durations and segment durations), in changes of the slope of the second formant (F2), and in decreases of acoustic vowel space. Yunusova et al. [31] used a 3D motion capture system to measure the kinematic movements of the jaw and lips during speech. They observed that changes in the path distance and speed of both lip and jaw movements anticipated the drop in speech intelligibility by approximately three months; and increases in the duration of these speech movements coincided with declines in speech intelligibility.

Although prior studies have identified promising subsystem markers of bulbar dysfunction, most of these studies were not designed to test their responsivity to the early stages of disease. Moreover, only a few studies have investigated the effects of the disease simultaneously on multiple speech subsystems in a small number of cases [8, 16]. Because persons with ALS vary in both the site of bulbar onset and the pattern of spread, the development of assessments that account for changes in all four major speech subsystems is essential.

In this study, we take a data-mining approach to achieve two aims: (1) deriving a set of quantitative subsystem predictors that are responsive to changes in bulbar motor function over the course of disease progression and (2) determining the time course of the changes occurring in multiple speech subsystems relative to the decline in system-level speech measures (i.e., speech intelligibility and speaking rate). We hypothesize that measures from the four major speech subsystems (i.e., respiratory, phonatory, articulatory, and resonatory) are individually and collectively more responsive than the system-level measures in detecting early changes in bulbar motor performance.

## 2. Materials and Methods

### 2.1. Participants

Sixty-six ALS patients (37 males and 29 females) aged from 39 to 79 years old (*M* = 57 years and SD = 10 years) participated in the study. All participants met the following criteria of recruitment: (1) were diagnosed with possible, probable, or definite ALS according to the revised El Escorial criteria; (2) spoke English as their primary language; (3) had no history of other congenital or acquired neurological disorders; (4) had normal hearing and vision adequate to read stimuli; (5) had literacy skills adequate to read the stimulus materials; (6) showed no signs of cognitive impairment as measured by the Montreal Cognitive Assessment (MoCA) (cut of score <26) [32]; and (7) were not on medications known to affect speech production [33].

Among all participants, 15 presented with bulbar onset, 41 with spinal onset, 6 with mixed bulbar and spinal onset of ALS, and 4 had an unknown onset site. Disease duration varied among participants. At the beginning of the study, patients were on average 12 months after diagnosis (SD = 18 months). The severity of ALS and its bulbar presentation, as measured by the Amyotrophic Lateral Sclerosis Functional Rating Scale-Revised (ALSFRS-R) [34], varied among participants at the beginning of the study as well. ALSFRS-R scores at the first visit ranged between 29 and 48, with a mean of 38 (SD = 5). The bulbar subscores, estimated based on the first 3 questions of the scale with a maximum score of 12, ranged between 4 and 12, with a mean of 10 (SD = 2).

The study was approved by the Ethics Research Boards at the Sunnybrook Research Institute in Toronto and University of Nebraska-Lincoln and was conducted with the understanding and the consent of the participants. All participants were recorded longitudinally over multiple sessions. Different numbers of sessions were recorded between participants, depending on the rate of disease progression. The average number of sessions across participants was 7 (SD = 5). The duration between the first and last sessions ranged from 1.4 month to 60 months (*M* = 15 months and SD = 12 months). The attempt was made to bring patients back every three months but the time between sessions varied due to the fact that the protocol was embedded into a clinical setting and the session schedule depended on the schedule of patient's return for clinical follow-up. In addition to significant dropouts between recordings, which is common for studies of ALS [35, 36], some patients were unable to complete the protocol in its entirety and thus contributed to missing data.

### 2.2. Data Acquisition: Materials and Measurements

For each participant, multiple variables were measured from each of the four speech subsystems (respiratory, phonatory, articulatory, and resonatory). The data for the subsystems were acquired using a variety of acoustic, aerodynamic, and kinematic instruments. A brief description of instrumentation, acquisition settings, and measurements is provided in the appendix and more detailed descriptions have been published previously by Yunusova et al. [37] and Green et al. [3].

#### 2.2.1. Respiratory Subsystem

The function of the respiratory subsystem was assessed using eight measures of speech pausing patterns and two measures of subglottal pressure (in /pa/ and /pi/) collected with the Phonatory Aerodynamic System (PAS) (KayPentax, USA). Pausing analyses have been used extensively to assess the communication deficits caused by neurologic impairments such as ALS [38, 39], traumatic brain injury [14], and Parkinson's disease [40] and showed sensitivity to disease-related respiratory changes in clinical populations. To evaluate speech pausing patterns, airflow was collected using a disposable mask that fitted around the participant's face while the participant was reading a standard 60-word paragraph developed specifically for accurate, automatic pause-boundary detection [38] at their normal comfortable rate and loudness. The airflow signal was then exported into a custom MATLAB program Speech Pause Analysis (SPA) [41], which identified the pauses in the signal, defined as silences longer than 300 milliseconds. To evaluate subglottal pressure, which assesses the integrity of the respiratory muscle function, the oral air pressure was recorded by positioning a pressure-sensing tube inside the participant's mouth when the participant was repeating a syllable (/pV/) seven times on one exhalation while maintaining consistent pitch and loudness. Peak oral air pressure was measured and averaged across the five middle repetitions of the syllable as estimations of subglottal pressure.

#### 2.2.2. Phonatory Subsystem

The function of the phonatory subsystem was assessed using 11 voice measures (e.g., jitter, shimmer, noise to harmony ratio [NHR], loudness, and maximum fundamental frequency [F0]) and one measure of laryngeal airway resistance. Voice measures have been used in previous studies to assess the lack of fine control in muscle tension and weakness in muscles involved in laryngeal valving and pitch change [20]. To obtain these measures, the acoustic signal was recorded using a high quality ear set microphone (Countryman E6) during a “normal” phonation of /a/ and a “high pitch” phonation of /a/, respectively. The “normal” phonation was produced by phonating /a/ at a normal pitch and loudness for 5 seconds. The “high pitch” phonation was produced by raising the pitch as high as possible from a normal pitch level and holding the phonation of /a/ at the highest pitch for up to 5 seconds. Three repetitions of each task were obtained. The acoustic signals were subsequently loaded into the Multidimensional Voice Profile (MDVP, Model 5105) software, in which (1) the middle 2 seconds of the “normal” phonation were used to derive the voice perturbation measures and (2) the stable interval of “high pitch” phonation where the pitch was at its highest was obtained to derive the maximum F0. Those who were not able to maintain their phonation for at least 2 seconds in both tasks were excluded from measurement. A variety of voice measures were obtained based on the selected interval of the signals. For example, maximum F0 range was determined as the difference between normal and highest F0 in the “high pitch” task. Laryngeal airway resistance, which assesses voicing efficiency [42], was obtained via PAS based on measures of peak air pressure, mean airflow, and mean sound pressure level (SPL) during “normal” phonation. Averages of the three repetitions were used in our analysis.

#### 2.2.3. Articulatory Subsystem

The function of the articulatory subsystem was assessed using 16 measures of upper/lower lip and jaw movements and three measures of alternating motion rate (AMR) in syllables. A high resolution 3D optical motion capture system was used to capture the positions of a set of reflective markers attached to the participant's forehead, vermilion border of the upper and lower lips, and three locations (left, middle, and right) on the chin while the participant was performing the assigned speech tasks (see Appendix). During postprocessing, maximum and minimum velocities of lip and jaw movements were obtained by (1) subtracting the head movement from the movements of lips and jaw; (2) loading the head-corrected lip and jaw movement trajectories into SMASH, a custom MATLAB program developed in our lab [43], to calculate the velocity as the first-order derivative of the corresponding movement trajectory; and (3) identifying the maximum and minimum velocities of lip opening (i.e., the relative movement between upper and lower lips), jaw movement, lower lip movement riding on the jaw, and lower lip movement relative to the jaw. These measures have been used in our earlier studies evaluating the effect of ALS on the articulatory kinematics [3, 31, 37]. The measures of alternating motion rate were obtained in a diadochokinetic (DDK) rate test in which the participant was asked to repeat /ba/ as clear and fast as possible on one breath. DDK rate assesses the overall oromotor ability of the participant to produce rapid and alternate speech movements. The number, duration, and rate of repetitions of the syllable were measured based on the acoustic signal.

#### 2.2.4. Resonatory Subsystem

Resonatory subsystem was assessed using 17 measures of velopharyngeal valving, which included peak oral air pressure and peak nasal airflow during oral and nasal consonants (/p/, /m/) embedded in different syllables, two measures of nasalance during sentence reading, and one measure of the lag between the peak nasal airflow during /m/ and the peak oral air pressure during /p/ in “hamper.” To obtain nasalance, which assesses velopharyngeal valving efficacy [44], a nasometer was used to collect the acoustic signals from the oral and nasal cavities, respectively, while the participant was reading “Buy Bobby a puppy” and “Mama made a lemon jam.” The intensities of the voiced portion of the oral and nasal acoustic signals were used to calculate nasalance, which is the ratio of nasal/nasal+oral acoustic energy, in the nasometer software. To obtain all other measures, the PAS was used, which collected nasal airflow by fitting a nasal mask around the participant's nose and recorded intraoral air pressure by placing a disposable pressure-sensing tube in the participant's mouth. Based on the recording, a variety of aerodynamic measures that assess velopharyngeal integrity including peak oral air pressure and peak nasal airflow during /p/ and /m/ and the lag between /m/ and /p/ in “hamper” were measured by the PAS software [45, 46].

#### 2.2.5. Speech System Measurement

In addition to the subsystem measurements, the Sentence Intelligibility Test (SIT) [47] was performed to obtain the system-level measurements of speech intelligibility and speaking rate. The SIT is a standard clinical approach to assess speech intelligibility in persons with motor speech disorders [47]. It has been used in our earlier studies [3, 31, 37] and has shown consistency with results reported in the ALS literatures [4, 9, 16].

During the test, participants were asked to read a list of 10 sentences of varying length (from 5 to 15 words) randomly generated by the SIT software. The speech samples of each participant were transcribed by one naive listener who was unfamiliar with either the test materials or the speech of the participants. Multiple listeners transcribed the speech samples over the time span of the study. Because the sentence list was randomly generated for each participant from a large inventory, it was unlikely that the same sentence was used frequently enough for the listeners to get familiar with it. Based on the SIT, speech intelligibility (i.e., the percent of words correctly transcribed out of the total number of words produced) and speaking rate (i.e., the number of words read per minute) were calculated automatically by the SIT software.

### 2.3. Data Reduction

To address the first aim of the study, which is to derive a set of quantitative subsystem predictors responsive to changes in bulbar motor function, we applied a data-driven approach that combined a principal component analysis (PCA) and linear mixed-effects (LME) models to the database comprising all measures as described above to reduce the number of measures to four comprehensive subsystem predictors (i.e., respiratory, phonatory, articulatory, and resonatory, resp.) of bulbar dysfunction.

#### 2.3.1. Variable Prescreening and Principal Component Analysis

A total of 61 subsystem variables (10 respiratory, 12 phonatory, 19 articulatory, and 20 resonatory) were recorded to comprise a multifactorial database. Recording a large number of variables is advantageous for deriving a data-driven statistical model of speech decline because speech is naturally a multifactorial event and because clinically-efficacious subsystem variables have not been fully identified. To eliminate the redundancy among the variables, we reduced the dimensionality of the database using a two-step variable screening procedure. First, the variables that were the most highly correlated with speaking rate were identified (*p* < 0.05 for Pearson's correlation). Speaking rate was used as the reference measure for subsystem variable screening because it is a more sensitive clinical predictor of bulbar dysfunction than speech intelligibility, which also declines linearly with disease progression unlike speech intelligibility [4]. As a result, 26 subsystem measures (6 respiratory, 2 phonatory, 12 articulatory, and 6 resonatory) were found to significantly correlate with speaking rate and were used in the second step of the analysis.

Second, to further reduce the dimensionality and eliminate intervariable correlations, the screened variables of each subsystem were subjected to a principal component analysis (PCA). PCA is a statistical procedure that uses an orthogonal transformation to convert a set of observations of possibly correlated variables into a set of linearly uncorrelated principal components [48]. Each principal component was comprised of a weighted sum of the subsystem variables, where the variables that determined the primary speech performance of the subsystem were assigned high weights. For each subsystem, a minimum set of principal components (PCs) that jointly accounted for over 95% of the total variance was selected to comprise a reduced database for further analysis.

#### 2.3.2. Linear Mixed-Effects (LME) Model

Because the principal components of each subsystem were linearly uncorrelated, their effects on bulbar performance were additive. To combine the principal components of each subsystem into a single predictor of the overall bulbar decline, we applied an LME model (*fitlme*, MATLAB R2013b) that predicted speaking rate as a function of the principal components of each subsystem and time (i.e., days after diagnosis) controlling for the subject effect. Because our data is a mix of within and between subject observations, the inclusion of a subject-dependent intercept as a random effect in the LME model accounted for the intersubject variations of speaking rate at the onset of data collection. Based on the LME models, a comprehensive predictor was derived for each subsystem as a weighted linear combination of the principal components using the beta coefficients of the fixed effect of the model. The subsystem predictors determined the contribution of each subsystem to speaking rate decline with respect to time.

### 2.4. Time until Speech Loss: Kaplan-Meier Analysis

**Figure 1:**
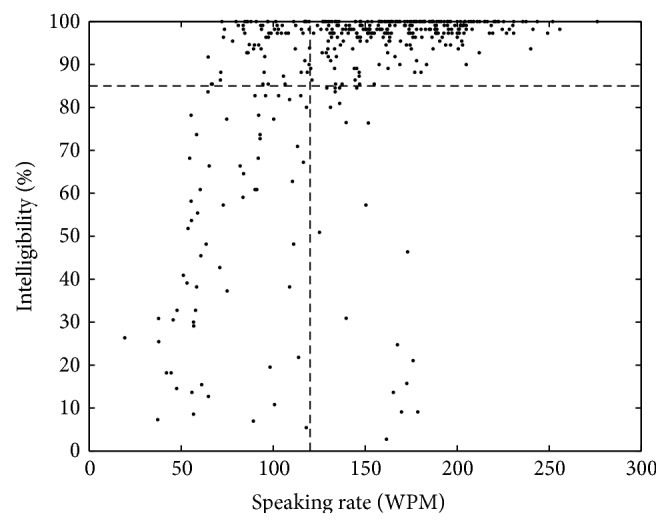
Scatter plot of the relation between speech intelligibility and speaking rate based on the SIT from all participants. The horizontal dashed line corresponds to 85% of intelligibility and the vertical dashed line corresponds to 120 WPM of speaking rate.

To address the second aim of the study, which is to determine the time course of the changes occurring in the speech subsystems relative to the declines in system-level speech measures, we applied a Kaplan-Meier survival analysis to estimate the time until speech loss, using the four subsystem predictors and two clinical speech measures (i.e., speech intelligibility and speaking rate) as estimators, respectively. The Kaplan-Meier analysis was originally developed to estimate the survival function from lifetime data and has been used widely in a variety of scientific fields (e.g., medical research, economics, engineering, ecology, etc.) to estimate the time course of the occurrence of an event [49]. In this study, we defined the time of speech loss as a critical event, which was characterized by slowing of speaking rate to 120 WPM. As discussed in the Introduction section, the slowing of speaking rate to 120 WPM marks the onset of rapid and substantial declines in bulbar speech function that result in the eventual loss of speaking ability. According to the relation between speech intelligibility and speaking rate as shown in Figure 1, the slowing of speaking rate to 120 WPM coincides with a decline of intelligibility to about 85%, which is consistent with previous findings [3, 4].

Because the Kaplan-Meier analysis provides a nonparametric estimation, it does not make assumptions on the distribution of data that are required by parametric methods such as linear regression. Therefore, using a Kaplan-Meier analysis to estimate the time course of bulbar speech decline is especially suited for a population as diverse as individuals with ALS.

We estimated the survival function, which was defined as the likelihood of maintaining speech function (i.e., speaking rate > 120 WPM), every 2.5 months within a 3-year time span after diagnosis using each of the six estimators (i.e., respiratory, phonatory, articulatory, and resonatory predictors, speech intelligibility, and speaking rate). Specifically, we first interpolated the values of each estimator at the specified intervals using a shape-preserving interpolation (*pchip*, MATLAB R2013b). Second, at each 2.5-month interval, we estimated the likelihood of maintaining speech function using different estimators by calculating the proportion of participants that (1) were more than 85% intelligible based on the SIT intelligibility score, (2) spoke faster than 120 WPM based on the SIT speaking rate, and (3) maintained respiratory/phonatory/articulatory/resonatory function to produce speech at a normal rate, which was characterized by a subsystem-based LME model prediction of speaking rate faster than 120 WPM. Based on the survival functions, visual comparisons were made between the subsystem and system-level estimators to evaluate their responsivity to bulbar decline during the disease progression.

## 3. Results

### 3.1. Principal Components of Subsystems

As described above, a set of principal components (PCs), which jointly accounted for over 95% variance, was determined for each speech subsystem. Two principal components (PCresp1 and PCresp2) accounted for 99.1% of the total variance in the respiratory subsystem. The key measures that comprised these PCs were the subglottal pressure during /pi/ and the number, duration, and frequency of pauses during passage reading. For the articulatory subsystem, three principal components (PCart1, PCart2, and PCart3) accounted for 96.5% of the total variance. These principal components were comprised of the following key measures: the maximum and minimum velocities of the lower lip movement riding on the jaw and the maximum and minimum velocities of lip opening in “Buy Bobby a puppy”, the maximum velocity of lower lip movement riding on the jaw and the maximum velocity of lip opening in /aCa/ (C is a consonant), and the number of syllable repetitions in the DDK test. Two principal components (PCreso1 and PCreso2) accounted for 99.5% of the variance in the resonatory subsystem. The key measures that comprised these PCs included the nasal airflow during /p/ and the mean nasalance in “Buy Bobby a puppy.” The key measures of each subsystem principal component are displayed in Table 1 along with their corresponding weights.

As for the phonatory subsystem, two principal components (PCphon1 and PCphon2) accounted for 100% variance because only two prescreened phonatory variables (i.e., maximum F0 and average laryngeal airway resistance) were subjected to PCA. However, the key measure (i.e., average laryngeal airway resistance) that comprised PCphon2 was only available for a relatively small number of participants, which limited the statistical power of the LME model. To determine whether PCphon2 must be included as a predictor of bulbar decline, we conducted a likelihood ratio test to compare an LME model with PCphon1 and time (i.e., days after diagnosis) as covariates with an alternative LME model with both PCphon1 and PCphon2 as well as time as covariates. We found no statistical difference between the two models (*p* = 0.59), so PCphon2 was dropped from the analysis. Meanwhile, because the average laryngeal airway resistance only had a minor effect on PCphon1, we replaced the missing values of this variable with zeros and updated PCphon1 to serve as the phonatory subsystem predictor of bulbar decline, which accounted for 77.7% of the variance in the phonatory subsystem.

### 3.2. Subsystem Predictors of Bulbar Decline

**Figure 2:**
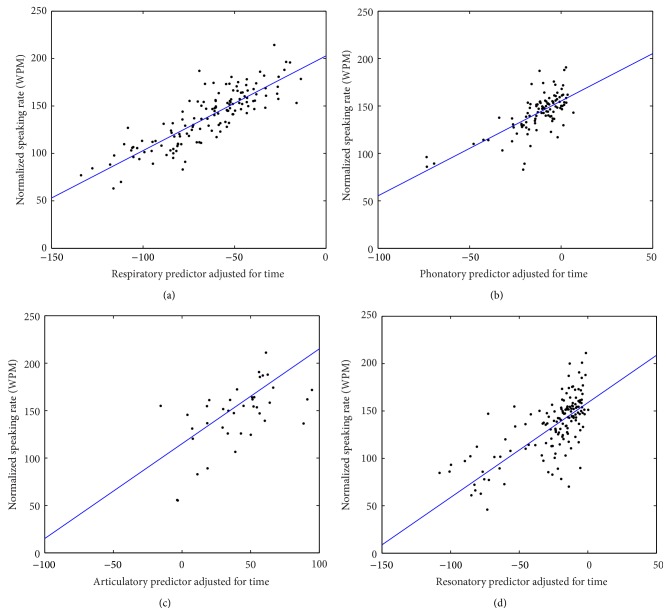
Scatter plots of the relation between speaking rate and each subsystem predictor accounting for time and subject effects: (a) respiratory, (b) phonatory, (c) articulatory, and (d) resonatory. On the *x*-axis is the subsystem predictor adjusted for time, which is the combination of each subsystem predictor and the effect of time on speaking rate. On the *y*-axis is the normalized speaking rate derived by subtracting the subject-dependent random effect from speaking rate. The lines are the linear fits based on the LME models.

Figure 2 shows scatter plots of normalized speaking rate against subsystem predictors accounting for time and subject effects. The subject effect (i.e., intersubject variability of speaking rate at the time of diagnosis) was accounted for by normalizing speaking rate through subtracting the subject-dependent random intercept of the LME model from the SIT speaking rate of each participant. The time effect (i.e., contribution of time to speaking rate drop in addition to the contribution of subsystems) was accounted for by adding its contribution to each subsystem predictor. The relatively high *R* ^2^ values in Table 2 and the goodness of fit in Figure 2 suggest the combination of subsystem predictors and time effect explained the bulk of variance in speaking rate decline.

### 3.3. Kaplan-Meier Estimation of Time until Speech Loss

**Figure 3:**
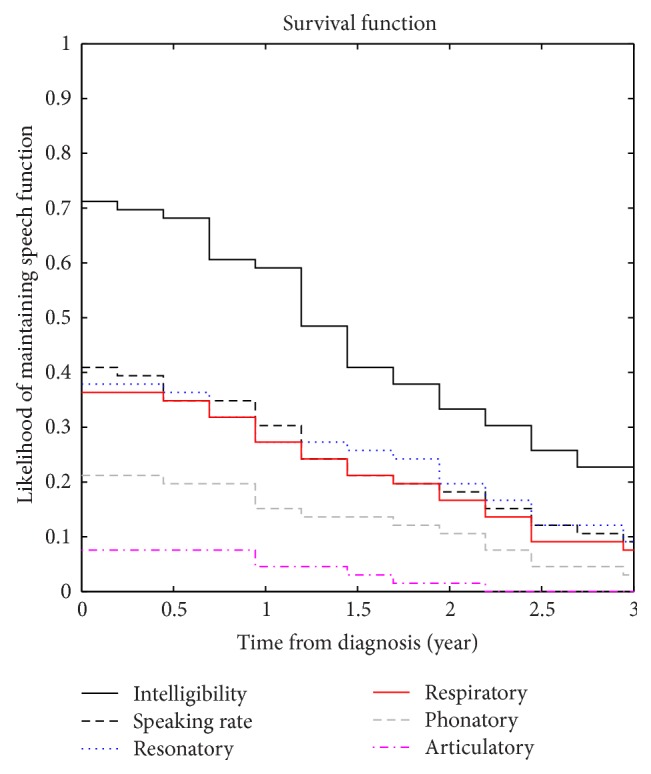
Survival functions for speech intelligibility (black solid), speaking rate (black dashed), respiratory (red solid), phonatory (grey dashed), articulatory (magenta dash-dotted), and resonatory (blue dotted) subsystem predictors.

Figure 3 shows the survival functions of four subsystem predictors and two system-level speech measures (speech intelligibility and speaking rate). By examining the survival functions at 2.5 months after diagnosis (i.e., first interval in Figure 3), we first compared the likelihood of maintaining speech function as estimated by the two system-level speech measures: (1) 71% of the participants had SIT intelligibility scores higher than 85% and (2) 41% of the participants had speaking rate faster than 120 WPM. Then we compared the likelihood of maintaining speech functions at 2.5 months after diagnosis as estimated by the four subsystem predictors: (1) 38% of the participants maintained resonatory function, (2) 36% of the participants maintained respiratory function, (3) 21% of the participants maintained phonatory function, and (4) 8% of the participants maintained articulatory function.

In three years after diagnosis (i.e., last interval in Figure 3), the likelihood of maintaining speech function was (1) below 10% as estimated by the articulatory and phonatory predictors, and (2) below 20% as estimated by the respiratory and resonatory predictors as well as by speaking rate, whereas there were still 30% of the participants with SIT intelligibility scores higher than 85%.

## 4. Discussion

The assessment of bulbar motor involvement is central to the diagnosis, prognosis, and the management of ALS. In this study, we used a data-driven approach to identify instrumentation-based measures of multiple subsystem functions that are sensitive to bulbar deterioration due to ALS. Different from most prior works, our approach incorporated information from multiple regions of the bulbar subsystem, which is essential because the onset of motor impairment and rate of decline can vary among different speech subsystems [8, 9, 50]. The current findings indicate that the declines in the articulatory and phonatory subsystems occur earlier than do changes in the standard clinical measures, suggesting that with further development, a subsystem approach will be advantageous for improving early detection and progress monitoring of bulbar diseases.

The data-driven approach identified several candidate variables from each speech subsystem that may underlie clinically discernable changes in speech. The observation that velocities of lip and jaw movement and velocities of lip opening were most susceptible to early bulbar decline among all subsystem measures is consistent with prior studies. Articulatory changes such as decreased extent and speed of jaw and lip motions and reduced contraction rate of tongue and lip muscles were observed during the early stages of the disease [8, 10, 31, 50–52]. Changes in speech kinematics might be attributed to weak, slow, and uncoordinated articulatory muscle activity, which eventually slows down speaking rate. The role of tongue kinematics in assessing bulbar dysfunction was not examined, although a number of previous studies suggested that lingual function was most affected by ALS among all articulators [3, 8, 10, 31]. The tongue function is the focus of our ongoing data collection and analyses and will be presented in future studies.

The results of the Kaplan-Meier analysis provided compelling evidence for the early involvement of articulatory motor function in the disease across most of our participants. Within 5 months after diagnosis, more than 90% of the participants showed substantial articulatory disturbances while only 60% of the participants had slowed speaking rates (<120 WPM) and only about 30% of the participants showed impaired intelligibility (<85%). As shown in Figure 3, about one year later, over 50% of the participants showed substantial drops in intelligibility as measured by SIT. This apparent delay between articulatory decline and speech intelligibility loss may afford speech-language clinicians the time, which is required to successfully transition patients to assistive communication devices. These findings motivate additional work that examines the value of articulatory markers for predicting later speech loss.

For the phonatory subsystem, the maximum F0 during the “high pitch” task was found to be responsive to early bulbar decline. This finding is consistent with previous studies that showed phonatory limits (e.g., maximum F0 range) declined over time, which served as a longitudinal sign of phonatory impairments due to ALS [20]. The observed limits in maximum vocal pitch suggest the presence of laryngeal valving inefficiencies [20] due to vocal fold weakness or spasticity, which can result in reduced subglottal airflow that necessitates more frequent inspirations and thus slows down speaking rate.

Regarding the respiratory subsystem, pause measures and subglottal pressure were observed to decline at a similar rate as speaking rate. The most likely explanation for this association is that one of the respiratory measures was pause duration, which is also a variable that determines the rate of speech [38–40]. Both measures are likely to be affected by respiratory muscle weakness [53] and the loss of fine motor control over the vocal tract musculature, where laryngeal and oropharyngeal weakness increases the resistance within the vocal tract, resulting in more frequent inspirations and a slower rate of speech. The poor responsivity of the respiratory subsystem to bulbar decline may have several explanations: (1) respiratory system is primarily innervated by spinal nerves rather than bulbar nerves; (2) persons with severe respiratory impairments were less likely to accomplish the required speech tasks, which might result in possible biases of our data towards persons with less impaired respiratory function; and (3) the indirect assessment of the respiratory function using speech breathing measures might be less sensitive than direct measures such as forced vital capacity (FVC). Previous studies suggested that reduced FVC is a reliable indicator of respiratory involvement in ALS [17, 54–56]. The relation between FVC and speech decline, however, has yet to be tested.

In the resonatory subsystem, nasal airflow during oral consonants and nasalance during sentence reading were found to correlate with changes in speaking rate. A comparison of the survival functions of the resonatory subsystem and speaking rate suggested that, at the onset and end of observation (i.e., 2.5 months and 3 years after diagnosis, resp.), the likelihood of resonatory decline and speaking rate drop was comparable; however, between 1.2 year and 2.4 years after diagnosis, the proportion of participants with slowed speaking rate exceeded the proportion of participants with resonatory decline. From these findings, we might infer that the resonatory predictor was less responsive to bulbar decline than was speaking rate between one and two years after diagnosis. The poor responsivity of the resonatory predictor may be, in part, because the slowing of speech allowed for oropharyngeal adjustments that confounded the effect of velopharyngeal inefficacy on the measures of nasal airflow and nasalance that comprised the resonatory predictor [57]. As disease progressed, however, oropharyngeal adjustments may become unavailable so that the confounding effect was removed, resulting in rapid declines in the resonatory measures regardless of the continuous slowing of speaking rate [45].

Although the resonatory and respiratory measures obtained in this study did not appear to be consistently affected across individuals early in bulbar ALS, there may be a subset of affected individuals for which these measures are efficacious markers of bulbar involvement. In addition, instrumental measures of the respiratory and resonatory subsystems other than those used in this study might be tested to evaluate their responsivity to bulbar decline. For example, future work is needed to evaluate the role of indirect measures of speech breathing with respect to the direct measures of respiratory function such as FVC, which is commonly used clinically.

While interpreting the responsivity of the subsystem measures to bulbar decline, we should acknowledge the covariation of some measures. Although the subsystem measures are assumed to assess the isolated status of the targeted speech subsystem, in practice, some of the measures are expected to covary because of acoustic, aerodynamic, or biomechanic dependencies within the vocal tract. For example, a slowing of oral opening due to muscular weakness has the potential to change nasal airflows [57]. Similarly, measures of the phonatory limits and the DDK rate may be affected by the respiratory function. Therefore, an important goal for future work is to design tasks that maximally isolate the performance of individual subsystems. For example, direct measures of the velopharyngeal function such as the movements of the velum might provide more sensitive indicators of resonatory involvement.

While assessing the sensitivity of subsystem measures to early bulbar decline with respect to system-level measures, we need to acknowledge that speech intelligibility is likely to be affected by both speaker and listener characteristics. The confounding effect from the listener on the assessment of speech function is common among perceptual speech tests and might be reduced by having multiple listeners perform the same task and conducting a reliability test. Despite the limitations related to the listener effect, our finding that the speech production system preserves function in the presence of subsystem impairments during the early stages of the disease is consistent with prior findings in ALS and other neurologic diseases [8, 9, 22]. More work is required to determine the extent to which, during the early stages of the disease, speakers modify speech subsystems control to maximize speech intelligibility [8].

Conducting longitudinal research in ALS is challenging. Due to the progressive nature of ALS, some participants lose the capacity to perform the required tasks and thus drop out from the study. As a consequence, the data from these participants are less robust due to a relatively small number of available observations across time. The use of an LME model that accounted for intersubject variability improved the robustness of the model of the subsystem performance in this study to some extent. In addition to the high dropout rate, some participants were unable to complete the protocol in its entirety during each visit due to factors such as fatigue, resulting in missing data across different subsystem measures. Although common in the ALS research, missing data could potentially bias the results. One possible remedy is to impute the predictor from each subsystem before applying the Kaplan-Meier analysis. We are working toward establishing imputation techniques that are suitable for our dataset.

Signs and symptoms at disease onset are often considered in studies of ALS. Bulbar onset is documented in up to 30% of patients with ALS, and as the disease progresses, almost all patients regardless of disease onset demonstrate bulbar involvement at later stages of the disease [58]. In this study, because the number of participants with bulbar onsets (i.e., 15 out of 66 participants) was not sufficient to perform a separate analysis, we combined the groups of participants with different onset sites to assess their bulbar dysfunction. Although combining participants with bulbar and spinal onsets might potentially introduce a confounding factor of severity (e.g., bulbar onset being more severe), the presentation of bulbar involvement does not seem to differ between the two groups. Therefore, we consider the findings of this study are not affected by disease onset.

## 5. Conclusions & Implications

Bulbar motor deterioration due to ALS was investigated using a data-driven approach based on commonly used clinical measures of speech decline (i.e., speech intelligibility and speaking rate) and instrumentation-based measures of the four major speech subsystems (i.e., respiratory, phonatory, articulatory, and resonatory). The findings showed that the subsystem measures that captured articulatory and phonatory dysfunction were affected prior to the presence of speech intelligibility deficits and the substantial slowing of speaking rate. Articulatory impairments including reduced lip and jaw movement velocities and reduced DDK rate as well as phonatory impairments such as reduced F0 range served as sensitive indicators of early bulbar decline. These findings suggest that (1) current assessment standards (i.e., speech intelligibility and speaking rate) may be inadequate for detecting bulbar involvement during the early stages of the disease and (2) monitoring changes in subsystem functions (especially, articulatory function) might help clinicians determine the timing of clinical intervention, including the implementation of augmentative and alternative communication (AAC). Follow-up work is needed to develop clinically feasible and standardized protocols that predict the rate of bulbar decline and the pattern of disease progression within an individual.

## Figures and Tables

**Table 1:** The key variables and the corresponding weights that comprise the principal components of each speech subsystem. The composite variables are spelt out below.

Principal components	Composite variables	Weights
PCresp1	SubGlotPrssMax_Pi	1.00

PCresp2	Pause_Event	0.51
	PerPause	0.76
	Pause_Duration	0.40

PCphon1	Max_F0	0.97

PCart1	BBP_MaxVel_LL_RBHo	−0.40
	BBP_MaxVel_UL_LLo	−0.56
	BBP_MinVel_LL_RBHo	0.40
	BBP_MinVel_UL_LLo	0.56

PCart2	APA_MaxVel_LL_RBHo	0.58
	APA_MaxVel_UL_LLo	0.79

PCart3	Reps_DDK	0.99

PCreso1	NasalFlow_Pi	1.00

PCreso2	Naso_BBP	1.00

*Notes*.

*SubGlotPrssMax_Pi* = peak intraoral pressure during /pi/.

*PerPause* = percent of pausing time.

*Pause_Event* = number of pauses.

*Pause_Duration* = duration of pauses.

*Max_F0* = maximum fundamental frequency during a high pitch task.

*BBP_MaxVel_LL_RBHo* = maximum velocity of lower lip movement riding on the jaw during “Buy Bobby a puppy.”

*BBP_MaxVel_UL_*LLo = maximum velocity of lip opening during “Buy Bobby a puppy.”

*BBP_MinVel_LL_*RBHo = minimum velocity of lower lip movement riding on the jaw during “Buy Bobby a puppy.”

*BBP_MinVel_UL_LLo* = minimum velocity of lip opening during “Buy Bobby a puppy.”

APA*_MaxVel_LL_*RBHo = maximum velocity of lower lip movement riding on the jaw during /aCa/.

APA*_MaxVel_UL_LLo* = maximum velocity of lip opening during /aCa/.

*Reps_DDK* = repetitions of syllable during the diadochokinetic rate test.

*NasalFlow_Pi* = peak nasal airflow during /pi/.

*Naso_BBP* = Mean nasalance score of the sentence “Buy Bobby a puppy.”

**Table 2:** Fixed effects and *R* ^2^ values for the LME models of the four subsystems (respiratory, phonatory, articulatory, and resonatory).

LME model	Fixed effect			*R* ^2^
	Intercept	Subsystem predictor	Time effect	
Respiratory	202.96	−0.14 ∗ PCresp1 − 1.7 ∗ PCresp2	−0.019 ∗ time	0.87
Phonatory	155.3	0.0072 ∗ PCphon1	−0.032 ∗ time	0.90
Articulatory	115.13	−0.079 ∗ PCart1 + 0.08 ∗ PCart2 + 0.2 ∗ PCart3	−0.036 ∗ time	0.89
Resonatory	158.8	−0.067 ∗ PCreso1 − 0.12 ∗ PCreso2	−0.028 ∗ time	0.86

**Table 3:** Instrumentation and data acquisition settings for measurements of speech subsystem functions.

Subsystem	Instrument	Signal	Task	Measurements
Respiratory	Phonatory Aerodynamic System (PAS), *KAYPentax *	Aerodynamic	/pa/, /pi/	Maximum subglottal pressure
			Bamboo passage	Speech duration, pausing pattern (e.g., number of pauses, pause duration, pausing frequency)

Phonatory	Compact flash recorder, professional quality microphone, *Countryman E6*, Phonatory Aerodynamic System, *KAYPentax *	Acoustic, aerodynamic	“Normal” and “high pitch” phonation of /a/	Phonation duration, maximum F0, jitter, shimmer, NHR, SPL, laryngeal airway resistance

Articulatory	Eagle Digital System, *Motion Analysis Corp. *	Kinematic	“Buy Bobby a puppy” “Say /aCa/ again”	Maximum/minimum velocities of lips and jaw
	Microphone	Acoustic	Repeat /ba/ as clear and as fast as possible on one breath	Number, duration, and rate of syllable repetitions

Resonatory	Nasometer, *Model 6400*, *KAYPentax *	Acoustic	“Mama made a lemon jam” “Buy Bobby a puppy”	Nasalance
	Phonatory Aerodynamic System, *KAYPentax *	Aerodynamic	/pa/, /pi/, /ma/, /mi/, “hamper”	Intraoral air pressure and nasal airflow in syllables, time lag between /m/ and /p/ in “hamper”

## Appendix

See Table 3.
